# Intramural Health Care Through Video Consultations and the Need for Referrals and Hospital Admissions: Retrospective Quantitative Subanalysis of an Evaluation Study

**DOI:** 10.2196/44906

**Published:** 2024-06-28

**Authors:** Katharina Schmalstieg-Bahr, Miriam Giovanna Colombo, Roland Koch, Joachim Szecsenyi, Friedrich Völker, Eva Elisabeth Blozik, Martin Scherer

**Affiliations:** 1 Department of General Practice and Primary Care University Medical Center Eppendorf Hamburg Germany; 2 A+ Videoclinic GmbH Gräfelfing Germany; 3 Institute for General Practice and Interprofessional Care University Hospital Tübingen Tübingen Germany; 4 Department of General Practice and Health Services Research University Hospital Heidelberg Heidelberg Germany; 5 Institute of Primary Care University Hospital Zurich Zurich Switzerland

**Keywords:** intramural health care, prison, telemedicine, primary care, family medicine, referral, hospital admission, admission rate, intramural, penal, video consult, e-consult, remote care, virtual care, health care delivery, service delivery, health care system

## Abstract

**Background:**

In comparison to the general population, prison inmates are at a higher risk for drug abuse and psychiatric, as well as infectious, diseases. Although intramural health care has to be equivalent to extramural services, prison inmates have less access to primary and secondary care. Furthermore, not every prison is constantly staffed with a physician. Since transportation to the nearest extramural medical facility is often resource-intensive, video consultations may offer cost-effective health care for prison inmates.

**Objective:**

This study aims to quantify the need for referrals to secondary care services and hospital admissions when video consultations with family physicians and psychiatrists are offered in prison.

**Methods:**

In 5 German prisons, a mixed methods evaluation study was conducted to assess feasibility, acceptance, and reasons for conducting video consultations with family physicians and psychiatrists. This analysis uses quantitative data from these consultations (June 2018 to February 2019) in addition to data from a sixth prison added in January 2019 focusing on referral and admission rates, as well as reasons for encounters.

**Results:**

At the initiation of the project, 2499 prisoners were detained in the 6 prisons. A total of 435 video consultations were conducted by 12 physicians (3 female and 7 male family physicians, and 2 male psychiatrists during the study period). The majority were scheduled consultations (341/435, 78%). In 68% (n=294) of all encounters, the patient was asked to consult a physician again if symptoms persisted or got worse. In 26% (n=115), a follow-up appointment with either the video consultant or prison physician was scheduled. A referral to other specialties, most often psychiatry, was necessary in 4% (n=17) of the cases. Only in 2% (n=8) of the consultations, a hospital admission was needed. Usually, hospital admissions were the result of unscheduled consultations, and the videoconferencing system was the method of communication in 88% (n=7) of these cases, while 12% (n=1) were carried out over the phone. Reasons for admissions were severe abdominal pain, hypotension, unstable angina or suspected myocardial infarction, or a suspected schizophrenic episode.

**Conclusions:**

Most scheduled and unscheduled consultations did not require subsequent patient transport to external health care providers. Using telemedicine services allowed a prompt patient-physician encounter with the possibility to refer patients to other specialties or to admit them to a hospital if necessary.

## Introduction

In 2022 approximately 56,000 people were incarcerated in a total of 172 German prisons. While 19,339 (34%) out of 56,557 inmates face imprisonment of over 1 year to 4 years, 5340 (9%) out of 56,557 inmates serve prison sentences of 5 years or longer [1,2]. By law, these prisoners have the same right to access health care as patients with statutory health insurance outside the correctional system. However, caring for incarcerated patients is challenging. They are more likely to experience alcohol or drug abuse, mental illnesses, or communicable diseases, such as hepatitis C or HIV infections [3,4], and not every prison is constantly staffed with a physician on-site. Specialized (secondary care) services are less available to inmates since a resource-intensive transport to the next extramural facility has to be organized. Telemedicine offers the potential to close this gap and improve intramural health care [5]. In this context, the collective term telemedicine describes heterogeneous concepts that aim at providing medical diagnostics, therapy, and rehabilitation despite physical distance or time lag [6]. In many countries, it has been used to facilitate or enhance intramural care [7-10], but it has not been implemented on a broad scale in the German correctional system yet.

This study aims to assess the need and the reasons for referrals to secondary care services, as well as hospital admissions, when scheduled or unscheduled telemedicine consultations with family physicians and psychiatrists are offered in prison.

## Methods

### Overview

The pilot project to establish video consultations in German prisons was initiated by the Ministry of Justice Baden-Württemberg in cooperation with A+ Videoclinic (VC), a provider of telemedical services, and initially, involved 5 German prisons. Between June and December 2018, the pilot project was evaluated in terms of feasibility and acceptance of the video consultations, as well as consultation reasons by conducting a mixed methods evaluation study. Quantitative and qualitative data were collected through site visits in the prisons, questionnaires, semistructured interviews, and consultation documentation. Further details are reported elsewhere [11]. This analysis is a retrospective subanalysis using a quantitative VC data set that was generated during the evaluation study period containing the information depicted in Table 1, as well as additional data from a sixth prison, which was not part of the initial pilot project.

All 6 prisons were located in the federal state of Baden-Württemberg, Germany. Inmates were male and female (adults and adolescents) and 18 years of age and older. Participation in the pilot study was voluntary. Patients could choose either a video consultation or regular medical care. If the patient opted for a video consultation, he or she had to sign an informed consent form.

Consultations that were conducted between June 2018 and February 2019 were analyzed in this study. The videoconferencing system (VCS) was the preferred method of communication. The phone was used in case of any technical problems with the VCS. Consultations were carried out by a team of 12 physicians (3 female and 7 male family physicians, and 2 male psychiatrists) employed by the telemedicine provider. Scheduled encounters were conducted during fixed weekly timeslots—either with VC-family physicians or a VC psychiatrist. Outside of these consultation hours, prison nursing staff could reach the on-call VC-family physician 24 hours 7 days per week. These patient-physician-contacts outside of consultation hours were counted as unscheduled consultations. Depending on the time of contact, there was not always a trained nurse present in prison. If the on-call family physician required help regarding a psychiatric problem, he or she could contact a VC psychiatrist.

The VC provided the telemedical infrastructure for VC physicians and prisons. Physicians documented the consultations electronically with a VC laptop using a virtual private network to access the VC software called Videoclinic Portal (Videoclinic) developed by the Videoclinic. The participating prisons were also equipped with VC laptops to receive the documentation. No remote medical devices, such as stethoscopes, that would have allowed the physician to directly auscultate a patient were used during the pilot study. Either prison nursing staff or correctional officers were present during each encounter. Prison nursing staff could obtain the patient’s vital signs (pulse, blood pressure, temperature, and oxygen saturation) and write an electrocardiogram if necessary and available. A total of 4 out of 6 prisons had an electrocardiogram on-site. Further details on the medical equipment available in the 5 prisons that were part of the initial pilot project can be found elsewhere [11].

For this study, an anonymized data set was exported from the VC software containing the variables depicted in Table 1. The data analysis for this study comprised descriptive statistical methods and was performed using Microsoft Excel 2016.

**Table 1:** Data provided by A+ Videoclinic.

Variable	Description
Date of consultation	Date and time of the encounter
Prison	Name of the prison
Physician	Treating physician (pseudonymized number D1-12)
Medical specialty	Family medicine Psychiatry
Assessment	Current assessment (free text)
Diagnosis	Current diagnosis (free text and International Classification of Diseases, 10th revision code)
Plan	Recommended treatment (free text)
Medication	Current medication if any prescribed (free text)
Type of consultation	Scheduled Unscheduled
Method of communication	Video Phone
Interpreter	Foreign language if interpreter was used
Follow-up	No further treatment Follow-up if symptoms persist or worsen Planned follow-up appointment Referral Hospital admission

### Ethics Approval

Ethics approval for the evaluation of the pilot project was obtained from the ethics committee of the Eberhard-Karls-University Tübingen (728/2018BO1) and the ethics committee of the State Medical Association Baden-Württemberg (F-2018-054) [11].

## Results

### Baseline Characteristics of the Participating Prisons

The ratio of prisoners to medical staff differed between the 6 prisons. Table 2 shows the sex and number of inmates of each prison at the initiation of the pilot study, as well as the number of physicians and nurses. Except for P4, prison staff comprised at least 1 physician. In P4, 2 external physicians offered scheduled consultation hours twice a week. Scheduled consultations with external physicians from other specialties (not family medicine) varied greatly. In most prisons, regular visits from a dentist were established. P2 offered appointments with a gynecologist and psychiatrist. In addition to dental care and the care provided by the prison physician, P6 offered consultations with a dermatologist and surgeon. Furthermore, nursing staff from P6 was able to contact an external psychiatrist if needed.

**Table 2:** Occupancy and staff per prison.

Prison	Characteristics	Number of prisoners (n=2499), n (%)	Number of physicians (n=12), n (%)	Number of nurses (n=69), n (%)
P1	Male adults	350 (14)	2 (17)	9 (13)
P2	Female adults or adolescents	350 (14)	1 (8)	6 (9)
P3	Male adolescents	395 (16)	4 (33)	9 (13)
P4	Male adults	52 (2)	0 (0)	3 (4)
P5	Male adults	772 (31)	4 (33)	22 (32)
P6	Male adults	580 (23)	1 (8)	20 (29)

### Referrals Following Video Consultations

From June 2018 to February 2019, VC physicians conducted 435 consultations. Out of that, 78% (n=341) of all consultations were scheduled, and the remainder were unscheduled (94/435, 22%). In 68% (n=294), patients were asked to consult a physician again if symptoms persisted or got worse. In 26% (n=115) a follow-up appointment with the video consultant or prison physician was scheduled. A referral to other specialties was necessary in 4% (n=17). The VCS was the method of communication in all 17 cases during which a referral was necessary. A total of 3 of these encounters were unscheduled. Patient transport to an extramural facility was indicated in none of these cases since the secondary care physician could either be contacted via video consultation, was able to come to the prison, or was employed by the prison but off duty during the initial encounter (Table 3).

Due to the small number of necessary referrals, it is hard to rank specialties based on the frequency of necessary referrals. The data showed mixed results with psychiatry as the most common specialty referred to (3/17, 18%), followed by urology (2/17, 12%) and proctology (2/17, 12%). In 2 cases, patients were referred to an external psychiatrist; and 1 patient had a follow-up appointment with a prison psychiatrist. Referral diagnoses also varied between the cases (Table 3). A total of 5 out of the 12 VC physicians (4 family physicians and 1 psychiatrist) conducted the encounters resulting in a referral, however, the number of overall encounters per physician varied greatly ranging from 2 to 143. An interpreter was not needed in any of the 17 consultations that resulted in a referral.

**Table 3:** Overview of the referrals to other specialties (n=17).

Type of consultation	Medical specialty (physician number)	Diagnosis	Medical specialty referred to
Unscheduled	FM^a^ (D7)	Drug abuse, opiate withdrawal	Psychiatry (coming to site)
Scheduled	PSY^b^ (D8)	Adjustment disorder	Child and adolescent psychiatry
Unscheduled	FM (D9)	Acneiform dermatitis	Dermatology (video consultation)
Scheduled	FM (D5)	Nausea, suspected adverse reaction, suspected melena	Rheumatology
Scheduled	FM (D10)	(missing data)	(missing data)
Scheduled	FM (D10)	Presbyopia	Ophthalmology
Scheduled	FM (D10)	(missing data)	(missing data)
Scheduled	FM (D5)	Ankle sprain R	Physician licensed to treat work accidents (German: *Durchgangsarzt*)
Scheduled	FM (D10)	(missing data)	(missing data)
Scheduled	FM (D5)	Adverse drug reaction	Urology
Scheduled	FM (D10)	Toothache	Dentistry
Unscheduled	FM (D9)	Prison admission exam	Psychiatry or drug counseling (prison psychiatrist)
Scheduled	PSY (D8)	Chronic arm pain after car accident, insomnia^c^	Neurology
Scheduled	PSY (D8)	Liver cirrhosis, suspected ascites^c^	Gastroenterology
Scheduled	FM (D5)	Priapism	Urology
Scheduled	FM (D5)	First degree hemorrhoids	Proctology^d^
Scheduled	FM (D5)	First degree hemorrhoids	Proctology^d^

^a^FM: family medicine.

^b^PSY: psychiatry.

^c^No diagnosis coded—information taken from medical history.

^d^Referral only necessary if symptoms persist despite ordered treatment.

### Hospital Admissions Following Video Consultations

In 2% (n=8) of the cases, a hospital admission was required. These cases were independent of the 17 cases that required a referral to another specialty in an ambulatory care setting. Hospital admission was usually the result of an unscheduled consultation (7/8, 88%), and the VCS was used in 88% (n=7). Gastrointestinal problems or pain were the most common reason for admission (4/8, 50%) and 6 VC physicians (5 family physicians and 1 psychiatrist) conducted the encounters (Table 4). An interpreter was not needed in any of these consultations.

**Table 4:** Overview of hospital admissions (n=8).

Type of consultation	Method of communication	Medical specialty (physician number)	Diagnosis
Unscheduled	Video	FM^a^ (D1)	Suspected tuberculosis, hypotension
Unscheduled	Video	FM (D7)	Unstable angina
Unscheduled	Video	FM (D1)	Severe hypotension suspected due to antipsychotics
Unscheduled	Video	FM (D11)	Gastrointestinal hemorrhage
Unscheduled	Phone	FM (D2)	Abdominal pain, kidney stones
Unscheduled	Video	FM (D2)	Gallbladder disease
Unscheduled	Video	FM (D4)	Abdominal pain (differential diagnosis: myocardial infarction)
Scheduled	Video	PSY^b^ (D8)	Suspected schizophrenia

^a^FM: family medicine.

^b^PSY: psychiatry.

### Hospital Admission and Referrals per Prison and Physician

Almost half of the video consultations (194/435, 45%) were conducted in prison P2 (Table 5). It was the only prison with female inmates (adults and adolescents). At the time of the initial visit to the participating prisons in the framework of the pilot project (September 2018), the number of inmates was similar to P1 (male adults) and P3 (male adolescents; Table 2).

A referral following a video consultation was needed in all prisons apart from P1 and P6. P3 had the highest number of referrals, but less than 10% of video consultations were conducted there. A hospital admission following a video consultation was required in all prisons except P3 and P4.

VC physicians D1 and D8 conducted more than 60% of all encounters (Table 6). However, referrals and admissions were rather scattered among physicians—except for D5, who initiated 6 (35%) of the referrals.

**Table 5:** Video consultations, referrals, and hospital admissions per prison.

Prison	Video consultations per prison, (n=435), n (%)	Referrals per prison, (n=17), n (%)	Hospital admissions per prison, (n=8), n (%)
P1	41 (9)	0 (0)	2 (25)
P2	194 (45)	5 (29)	1 (13)
P3	41 (9)	6 (35)	0 (0)
P4	75 (17)	4 (24)	0 (0)
P5	68 (16)	2 (12)	4 (50)
P6	16 (4)	0 (0)	1 (13)

**Table 6:** Video consultations, referrals, and hospital admissions per physician.

Physician	Video consultations per physician, (n=435), n (%)	Referrals per physician, (n=17), n (%)	Hospital admissions, (n=8), n (%)
D1	143 (33)	1 (6)	2 (25)
D2	9 (2)	0 (0)	2 (25)
D3	5 (2)	0 (0)	0 (0)
D4	18 (4)	0 (0)	1 (13)
D5	14 (3)	6 (35)	0 (0)
D6	8 (2)	0 (0)	0 (0)
D7	2 (1)	1 (6)	1 (13)
D8	124 (29)	3 (17)	1 (13)
D9	9 (2)	2 (12)	0 (0)
D10	97 (22)	4 (24)	0 (0)
D11	3 (1)	0 (0)	1 (13)
D12	3 (1)	0 (0)	0 (0)

## Discussion

### Principal Findings

Most scheduled and unscheduled video consultations did not require a subsequent patient transport to an extramural health care provider or facility. A referral was only needed in 4% (n=17) of the cases and hospital admission was only required in 2% (n=2) of 435 cases.

### Comparison to Prior Work

To our knowledge, this is the first study that focused on referral and hospital admission rates in intramural health care when video consultations with family physicians and psychiatrists are offered. Another study evaluated telemedicine consultations with an emergency room (ER) physician and found that 36% of the patients from a correctional facility required transport to the ER after a video encounter [12]. This rate was higher than the referral and hospital admission rate of this study. If prison physicians were given the possibility to refer patients to a telemedicine satellite facility for subspecialty consults, outpatient visits would increase by 40% in the 2 years after implementation. In contrast, ER visits decreased. The authors interpreted the effect as better access to care [13]. The same authors examined telemedicine programs in the juvenile justice system by measuring, for example, outpatient costs, ER costs, and transportation costs [14], which are parameters that are associated with referrals and admissions. Other studies focused on the treatment of single diseases, such as hepatitis C or diabetes, when telemedicine consultations were offered in prison [15,16].

Other studies have shown that outside of the correctional system home telemonitoring could reduce hospital admission rates in people with chronic obstructive pulmonary disease [17,18] and congestive heart failure [19,20]. Telemedicine consultations with an emergency physician led to reduced transfers from skilled nursing facilities to ERs and subsequently to lower hospital admission rates [21], and Rosner et al [22] showed that using telemedicine reduced readmissions after hip and knee arthroplasties. In contrast, a meta-analysis that evaluated different remote management strategies for patients with inflammatory bowel disease demonstrated a reduction in physician visits but no significant effect on relapse or hospital admission rates [23].

Regarding a reduction of referral rates, previous studies outside of the correctional system generally focused on electronic solutions for primary care providers to contact other specialists and have demonstrated mixed results. Liddy et al [24] reported that implementing an electronic consultation service in Canada that allowed family physicians to communicate with secondary care providers regarding a patient’s care reduced referral rates between 36% and 53%, which was in line with prior findings of that group [25]. However, their randomized controlled trial with 2 study arms (primary care physicians with and without access to the electronic consultation service), showed a significant referral reduction in both arms [26]. Furthermore, according to an online survey among primary care physicians, a phone consultation with an HIV specialist reduced the perceived need to refer the patient to a secondary care provider, although the authors acknowledged that actual referral rates had not been studied [27]. In another study, web-based consultation between primary care physicians and nephrologists did not affect referral rates [28].

In comparison to referral rates of (extramural) family medicine practices without the use of video consultations, a referral rate of 3.8% found in this study is at the lower end of the expected spectrum. Generally, referral rates to secondary care providers vary between practices. A recent study showed an average monthly rate of 20.3% with a range of 0.4%-67.1%. Outside of the correctional system, mental health services were the 10th most common specialty for referrals [29]. Older studies showed mean rates of 1.4% to 37% [30-33]. Variance can also be found regarding hospital admission rates of (extramural) family medicine practices. Mean rates of approximately 50-53 admissions per 1000 patients per year have been reported [34,35], which can only indirectly be compared with the data of this analysis (8 admissions per 435 encounters within 9 months).

International studies showed that telemedicine was able to deliver high-quality and timely primary care for adult and adolescent prison inmates [13,36], reduce costs [37], and facilitate mental health services [38,39]. Using telemedicine to improve access, cost, and quality of secondary care, for example, in the fields of ophthalmology [40,41], cardiology [42], and dentistry [10,43] has also been described before and is still under evaluation [44]. Especially, countries with remote regions outside of metropolitan areas, such as the United States or Australia, have reported the use of telemedicine in their correctional systems [45], but also more densely populated countries deemed the use as beneficial [10,46].

### Strengths and Limitations

The pilot project was the first broad implementation of telemedicine in the German correctional system. But there are some limitations: first, a possible selection bias has to be considered: prison staff may have chosen to directly call an ambulance or organize a transport to an extramural facility instead of using video consultations for more severe medical cases. If only patients with less severe diseases were seen by VC physicians, the likelihood of required hospital admission was consequently decreased. Similarly, prison staff may have directly scheduled an appointment with a secondary care provider visiting the prison, which also might have reduced the likelihood of referrals. However, it was crucial that not only patients were given a choice to talk to a VC physician or receive usual care, but that prison staff was also free to choose whether or not to contact the VC—especially since 2 authors were VC founding members. Neither prison staff nor patients received incentives for contacting the VC. Second, there was no control group in this study, and therefore, referral and hospital admission rates cannot be compared with regular intramural care. Despite the low rates when using video consultations, a referral or an admission might not have been necessary if an in-person consultation had been done at that time. Third, only data from the VC portal was considered for this analysis. Fourth, it is unknown how many patients refused a video consultation. Fifth, at the beginning of the data collection, some VC physicians did not complete the entire documentation template, therefore, some data were missing (the type of follow-up was not specified in 1 case, and in 2 cases, no diagnosis or reason for the referral was listed). The documentation improved throughout the project as physicians became more familiar with or received more training regarding the use of the VC portal. Finally, all consultations were carried out by a rather small group of physicians, and therefore, the influence of individual experience and working style cannot be excluded.

### Implication for Practice and Research

The results show that a referral or admission was only required in a few cases after video consultations were offered in prison. Compared with extramural family medicine practices, the referral rates found in this study were at the lower end of the expected spectrum and support that the implementation of telemedicine in intramural systems on a larger scale should not be postponed or revised due to concerns of high referral and admission rates. Further research, including controlled studies, is needed to explore whether institutional factors contribute to the effectiveness and safety, such as training of staff, use of remote medical devices, and acceptance of telemedicine by inmates, prison (nursing) staff, and physicians working on-site—especially in the light of the fact that the data were generated prior to the COVID-19 pandemic and video consultations became much more common since then.

## Abbreviations

ER: emergency room
VC: video clinic
VCS: videoconferencing system

## Data Availability

The data sets generated or analyzed during this study are not publicly available due to privacy protection regulations but are available from the corresponding author on reasonable request.
